# Determination of RNA polymerase binding surfaces of transcription factors by NMR spectroscopy

**DOI:** 10.1038/srep16428

**Published:** 2015-11-12

**Authors:** Johanna Drögemüller, Martin Strauß, Kristian Schweimer, Marcel Jurk, Paul Rösch, Stefan H. Knauer

**Affiliations:** 1 Lehrstuhl Biopolymere und Forschungszentrum für Bio-Makromoleküle, Universität Bayreuth, Universitätsstraße 30, 95447 Bayreuth, Germany

## Abstract

In bacteria, RNA polymerase (RNAP), the central enzyme of transcription, is regulated by N-utilization substance (Nus) transcription factors. Several of these factors interact directly, and only transiently, with RNAP to modulate its function. As details of these interactions are largely unknown, we probed the RNAP binding surfaces of *Escherichia coli* (*E. coli*) Nus factors by nuclear magnetic resonance (NMR) spectroscopy. Perdeuterated factors with [^1^H,^13^C]-labeled methyl groups of Val, Leu, and Ile residues were titrated with protonated RNAP. After verification of this approach with the N-terminal domain (NTD) of NusG and RNAP we determined the RNAP binding site of NusE. It overlaps with the NusE interaction surface for the NusG C-terminal domain, indicating that RNAP and NusG compete for NusE and suggesting possible roles for the NusE:RNAP interaction, e.g. in antitermination and direct transcription:translation coupling. We solved the solution structure of NusA-NTD by NMR spectroscopy, identified its RNAP binding site with the same approach we used for NusG-NTD, and here present a detailed model of the NusA-NTD:RNAP:RNA complex.

Transcription of genomic information from DNA to RNA is the initial step in gene expression, with RNA polymerase (RNAP) being the key enzyme of this process in all domains of life^1^. Bacterial core RNAP consists of five subunits, 2x α, β, β′, and ω. While the α subunits promote the assembly of the enzyme and are target of many regulatory proteins^2^,^3^,^4^, the β and β′ subunits form the active site and catalyze RNA synthesis^5^,^6^. The ω subunit is supposed to play a structural rather than a functional role. It binds to the N- and C-termini of the β′ subunit to prevent β′ aggregation until the ωβ′ complex is integrated into the RNAP^7^. During initiation of transcription the σ factor binds to core RNAP to form the holo enzyme, and σ is also essential for the recognition and melting of promoter regions (reviewed in^8^). The transcription cycle consists of three major phases: initiation, elongation, and termination. It is highly regulated by a multitude of transcription factors that bind to RNAP modifying its action. Prominent examples are the N utilization substance (Nus) factors that influence especially elongation and termination. Among all transcription factors NusG (Spt5 in archaea and eukaryotes) is unique as it is the only one that is universally conserved^9^. *Escherichia coli* (*E. coli*) NusG is a two-domain protein, with an N-terminal domain (NTD) and a C-terminal domain (CTD) connected *via* a flexible linker^10^. During elongation NusG-NTD binds to RNAP, enhancing the elongation rate and suppressing pauses^10^,^11^. To fulfill this function NusG-NTD contacts the β′ clamp helices (β′CH) and the β gate loop (βGL), closing the active site cleft so that the nucleic acids are locked and the transcription elongation complex (TEC) is stabilized (Fig. 1)^12^,^13^. Although NusG/Spt5-NTDs highly likely have the same function in all domains of life, NusG/Spt5-CTDs are targets of various interaction partners and thus serve as recruitment platform for further accessory factors. In *E. coli*, NusG-CTD binds to the termination factor Rho, promoting Rho-dependent termination^14^,^15^. Additionally, *E. coli* NusG-CTD interacts with ribosomal protein S10 to couple transcription and translation (Fig. 1)^14^. S10 is identical to transcription factor NusE that forms a complex with NusB and as such is involved in antitermination^16^. In the multiprotein antitermination complex RNAP is modified to be able to read through termination signals, a process that is essential for efficient transcription of ribosomal RNA operons^17^ or the DNA of lambdoid phages^18^. The NusE:NusB complex formed during antitermination binds to the single stranded, highly conserved *BoxA* RNA sequence^19^ and is anchored to RNAP *via* NusE:NusG-CTD interaction^14^. However, NusE is also able to bind directly to RNAP where it remains during elongation^16^,^20^. This interaction may be involved in antitermination, and the binding site on RNAP is suggested to be located in the β subunit^20^.

NusA is a multidomain protein consisting of an NTD, an S1, and two K-homology RNA binding domains, KH1 and KH2, the latter three forming the SKK domain. In *E. coli* and several other proteobacteria the NusA C-terminus comprises two acidic repeat domains, AR1 and AR2^21^,^22^. With its multitude of interaction partners, NusA is able to accomplish various functions. It modulates Rho-dependent and intrinsic termination, it either prolongs pauses or introduces new ones, and it is part of the antitermination complex (reviewed in^23^,^24^). NusA interacts directly with RNAP *via* NusA-NTD and NusA-AR2 (Fig. 1)^25^. While a high resolution solution nuclear magnetic resonance (NMR) structure is available for the complex of NusA-AR2 and the CTD of the RNAP α subunit (α-CTD)^4^, the RNAP interaction surface of NusA-NTD is not experimentally defined in atomic detail. A low resolution electron microscopy structure of the *Bacillus subtilis* (*B. subtilis*) NusA-NTD:RNAP complex as well as initial binding models are available and all studies suggest that NusA-NTD binds to the flap region of the β subunit at the RNA exit channel^26^,^27^,^28^. However, the exact RNAP binding surface on NusA-NTD remains to be determined.

Knowledge of the RNAP interaction surfaces of transcription factors is crucial for the complete understanding of RNAP regulation. Owed to the molecular mass of RNAP (*E. coli* RNAP ~390 kDa), the main techniques to study RNAP:transcription factor complexes structurally in atomic detail are X-ray crystallography and electron microscopy. However, RNAP regulation heavily depends on transient interactions and dynamics, i.e. information not easily accessible by these techniques. Thus, we chose to study *E. coli* RNAP:Nus factor interaction by NMR spectroscopy to identify the RNAP binding surface of these transcription factors. Our approach is based on observations that even in systems >100 kDa methyl groups are excellent NMR probes as they are still mobile enough to produce highly resolved spectra with good signal intensities owed to their fast motions around the methyl axis^29^.

## Results and Discussion

### RNAP interface of NusG-NTD

To identify the RNAP binding surface of transcription factors the methyl groups of Ile (δ1), Leu (δ1 or δ2), and Val (γ1 or γ2) residues of the respective, deuterated factor were labeled with [^1^H,^13^C] ([I,L,V]-labeled transcription factor; for clarity, all protein names without prefix refer to *E. coli* proteins). The titration of this [I,L,V]-labeled regulator with protonated RNAP was observed by two-dimensional (2D) [^1^H,^13^C]-methyl transverse relaxation optimized spectroscopy (TROSY). As a test case for the applicability of this method, we asked whether we were able to confirm the RNAP binding surface of NusG-NTD. This surface is known from a crystallographic study of the archaeal Spt4/5 complex with the β′ clamp domain of RNAP and biochemical experiments on NusG and RfaH, the latter being a paralog of NusG^12^,^13^.

**Figure 2:**
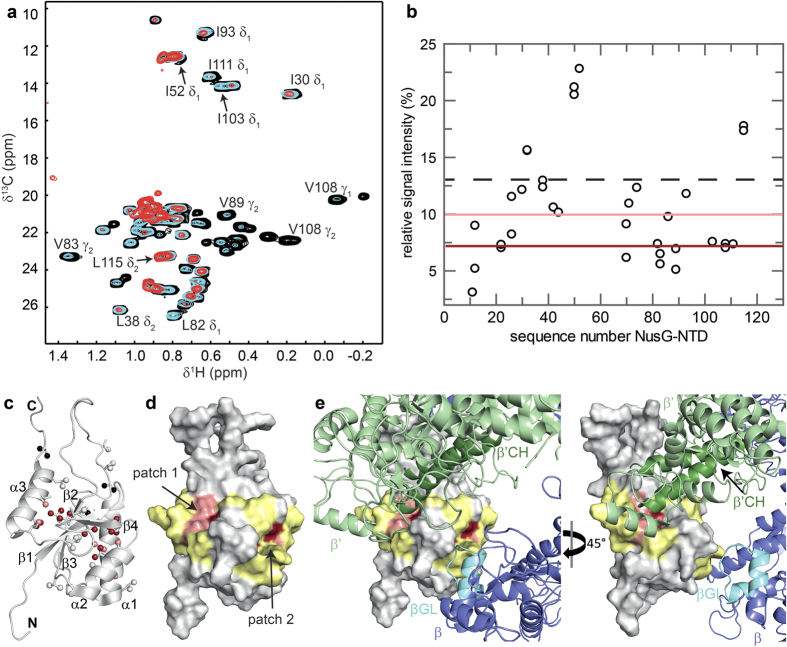
RNAP binding site of NusG-NTD. (**a**) Titration of [I,L,V]-NusG-NTD with protonated RNAP. Methyl-TROSY spectra of [I,L,V]-NusG-NTD in the absence, black, and in the presence of RNAP (1:1 molar ratio, cyan; 1:2 molar ratio, red). Selected signals are labeled. (**b**) Relative signal intensity of [I,L,V]-NusG-NTD after addition of RNAP in equimolar concentration *vs*. residue number of NusG-NTD. The dashed black line indicates the average relative signal intensity. Dark red and light red lines indicate the thresholds for strongly affected (55% of the average relative intensity) and slightly affected (75% of the average relative intensity) methyl groups, respectively. (**c**) Mapping of affected methyl groups onto the NusG-NTD structure (Protein Data Bank (PDB) ID: 2K06, cartoon representation, grey). Ile, Leu, and Val residues are in stick representation with the carbon atoms of their methyl groups as spheres. Strongly affected methyl groups, dark red; slightly affected methyl groups, light red; unaffected methyl groups, grey; unassigned methyl groups, black. Secondary structure elements and termini are labeled. (**d**) Mapping of affected residues onto the NusG-NTD structure (surface representation). For graphical illustration of the interaction site the complete amino acid was colored as affected in lieu of the methyl group. Colors are as in (**c**). Two amino acids on either side of affected Ile/Leu/Val residues are highlighted in yellow unless they were unaffected Ile/Leu/Val residues. (**e**) Model of NusG-NTD as in (**d**) bound to *E. coli* RNAP (PDB ID: 4KMU). The model is based on the structure of the *Pyrococcus furiosus* (*P. furiosus*) Spt4/5 complex bound to the RNAP clamp domain (PDB ID: 3QQC). NusG-NTD was superposed on Spt5 and RNAP β′ subunit on the clamp domain. As NusG-NTD and RNAP were treated as rigid bodies and no further optimization was carried out some minor clashes occur. β subunit, light blue; β′ subunit, light green; β′CH, dark green; βGL, cyan.

Upon addition of RNAP, the methyl group signals of [I,L,V]-NusG-NTD decreased in intensity, but not uniformly over all signals (Fig. 2a), likely caused by a combination of several effects. First, a general loss of signal intensity is owed to [I,L,V]-NusG-NTD:RNAP complex formation as the molecular mass (MM) of the complex is roughly 30-fold that of [I,L,V]-NusG-NTD (MM_NusG-NTD_ = 14 kDa, MM_RNAP_ = 389 kDa), resulting in severe line broadening. Second, by binding of [I,L,V]-NusG-NTD to RNAP, the specifically labeled methyl groups of [I,L,V]-NusG-NTD located in the binding interface get into close proximity of the RNAP protons, and the resulting intermolecular dipole-dipole interactions cause an additional contribution to relaxation, so that the signal intensity of methyl groups in the binding surface decreases more strongly than that of methyl groups located elsewhere in [I,L,V]-NusG-NTD. Finally, signal intensities can be influenced by chemical exchange processes in the intermediate range of the NMR timescale. Quantitative analysis of signal intensities for the 1:1 complex revealed two patches in the protein structure where signal intensities changed noticeably (Fig. 2b,c). Patch 1 comprises residues in helix α3 and strands β1 and β3, while patch 2 is formed by residues located in helices α1 and α2, and these two patches are located at nearly opposite sides of NusG-NTD. No assigned, but unaffected methyl groups were found in either of these patches. This approach provides only information about Ile, Leu, and Val residues, but most likely additional amino acids, especially in the direct vicinity of the affected residues, are involved in the interaction. Thus we graphically extended the representation of patches 1 and 2 by including the two residues preceding and following each affected Ile, Leu, or Val residue, unless they were unaffected Ile, Leu, or Val residues, resulting in two continuous regions (Fig. 2d). In a model of NusG-NTD bound to RNAP based on the crystal structure of the archaeal Spt4/5: β′ clamp domain complex^12^, residues of patch 1 are in direct proximity of the β′CH, indicating that we identified correctly the β′CH binding site (Fig. 2e). The NTD of RfaH, an *E. coli* paralog of NusG, not only interacts with the β′CH, but also binds to the βGL *via* His65, Thr66, and Thr67 which form an HTT motif located at the N-terminus of helix α2 (Supplementary Fig. 1)^13^. Although this interaction does not contribute significantly to the overall affinity of RfaH-NTD for RNAP it is essential for the antipausing activity of RfaH^13^. Similarly, structurally homologous residues in NusG-NTD (Ser79-His81) have been proposed to be involved in βGL binding, suggesting that this interaction is a general feature of NusG-like proteins^13^. NusG-NTD patch 2 corresponds to the RfaH region that is in immediate neighborhood of the βGL binding motif suggested for RfaH-NTD (Supplementary Fig. 1)^13^. Due to the absence of Ile, Leu, and Val residues in the NusG-NTD region that is structurally homologous to the HTT motif in RfaH, no direct information about this region is available in our experiments (Supplementary Fig. 1). Thus, we conclude that either the βGL binding surface in NusG-NTD differs slightly from the one in RfaH-NTD or that patch 2 constitutes only part of the βGL interaction surface or that residues of patch 2 are indirectly affected as they are located next to the actual binding site.

The clamp domain undergoes structural rearrangements during the transcription cycle, having closed and open conformations, and NusG-NTD/RfaH-NTD is proposed to lock the clamp in a closed state during elongation by making bridging contacts between the β′CH and the βGL so that the downstream DNA is completely encircled^13^,^30^,^31^,^32^,^33^. Hence, the elongation complex is stabilized and structural rearrangements that occur during pausing are prevented, which, in turn, leads to increased processivity. As we used core RNAP in our experiments the clamp is probably in an open state. Thus our findings indicate that in the absence of nucleic acids NusG-NTD contacts the β′CH and βGL either separately or simultaneously, suggesting that the RNAP claw is in a conformation that allows these contacts or that NusG-NTD induces a closed state.

Overall, the binding surfaces identified here are consistent with the previously published interaction sites of NusG-NTD, demonstrating that the present approach may be used to determine the RNAP binding surfaces of transcription factors in solution in a single experiment using intact RNAP and avoiding molecular alteration of the constituents. However, the limited number of NMR probes and their distribution over the structure restricts the structural resolution of the resulting binding site. Although we are not able to distinguish between methyl groups that are directly involved in the molecular interaction from those that are only indirectly affected, the careful interpretation of the surface representation allows us to identify the interaction surface.

### RNAP interface of NusE

Transcription factor NusE/S10 not only interacts with RNAP *via* NusG, but it is also able to bind directly and specifically to the RNAP β subunit during transcription^14^,^16^,^20^. The function of this interaction is still unknown. In order to study the molecular details of this interaction we determined the RNAP binding surface of NusE with the same approach as for NusG-NTD. As NusE alone is very unstable and tends to aggregate we used a NusE variant that lacks the ribosome binding loop (NusE^Δ^) in complex with NusB for our experiments^34^. The presence of NusB does not influence the NusE^Δ^:RNAP interaction^20^. For the NMR titration, we labeled the methyl groups of Ile, Leu, and Val residues of NusE^Δ^ in the deuterated NusB:NusE^Δ^ complex with [^1^H,^13^C] ([I,L,V]-NusE^Δ^).

**Figure 3:**
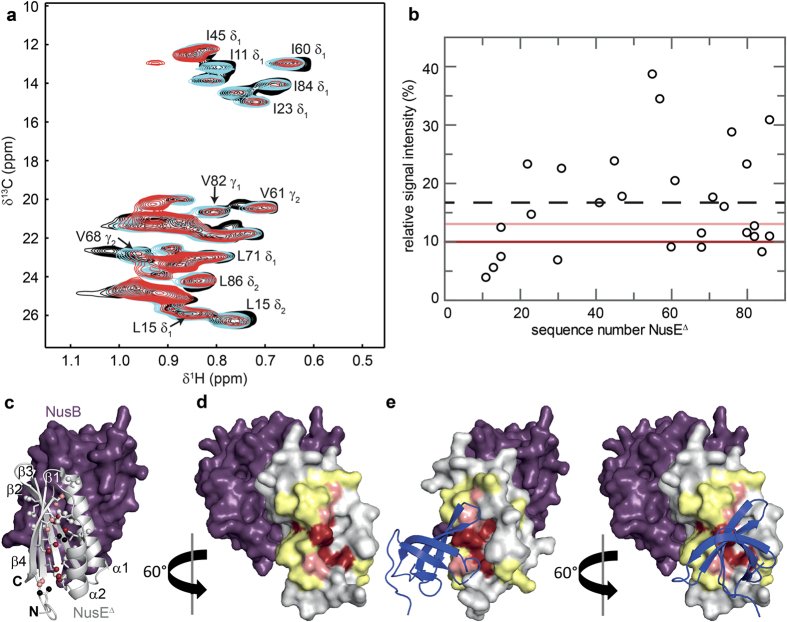
RNAP binding site of NusE^Δ^. (**a**) Titration of [I,L,V]-NusE^Δ^ with protonated RNAP (NusE^Δ^ being in complex with deuterated NusB). Methyl-TROSY spectra in the absence, black, and in the presence of RNAP (1:1 molar ratio, cyan; 1:2 molar ratio, red), with representative signal assignments. (**b**) Relative [I,L,V]-NusE^Δ^ signal intensity after addition of RNAP in a 1:2 molar ratio *vs*. amino acid sequence positions of NusE^Δ^. Dashed black line, average relative signal intensity; dark red and light red lines, thresholds for strongly affected (60% of the average relative intensity) and slightly affected (80% of the average relative intensity) methyl groups, respectively. (**c**) Mapping of affected methyl groups onto the NusB:NusE^Δ^ complex structure (PDB ID: 3D3B; NusB, purple; NusE^Δ^, light grey). NusB in surface, NusE^Δ^ in cartoon representation. Ile, Leu, and Val residues in NusE^Δ^ are represented as sticks with the carbon atoms of their methyl groups as spheres. Strongly affected methyl groups, dark red; slightly affected methyl groups, light red; unaffected methyl groups, grey; unassigned methyl groups, black. Secondary structure elements and termini are labeled. (**d**) Mapping of affected residues onto the NusB:NusE^Δ^ complex structure (surface representation). Colors are as in (**c**). For graphical illustration of the interaction site the complete amino acid was colored as affected in lieu of the methyl group. Two amino acids on either side of an affected Ile/Leu/Val residue are highlighted in yellow unless they were unaffected Ile/Leu/Val residues. (**e**) Structure of the NusB:NusE^Δ^:NusG-CTD complex. The NusE^Δ^:NusG-CTD complex (PDB ID: 2KVQ, NusG-CTD in blue cartoon representation) was superposed on the NusB:NusE^Δ^ complex from (**d**).

Upon addition of protonated RNAP, [I,L,V]-NusE^Δ^ methyl group signals decreased in varying proportion (Fig. 3a,b). All highly and slightly affected methyl groups are located in helices α1 and α2 as well as strands β1 and β4 (Fig. 3c). Inspection of the surface representation and the graphical extension as carried out for NusG-NTD result in a continuous patch (Fig. 3d). As the 7 Ile, 10 Leu, and 7 Val residues of NusE^Δ^ (86 residues overall) are distributed evenly over the sequence and the structure, our definition of the interaction surface is highly reliable. The RNAP binding site is opposite of the NusB:NusE^Δ^ interface and the ribosome integration site, i.e. the NusE^Δ^:RNAP interaction is not only possible within the context of the NusB:NusE^Δ^ complex, but also when NusE is integrated into the ribosome^35^. NusE could thus simultaneously accommodate the ribosome and the RNAP.

**Figure 4:**
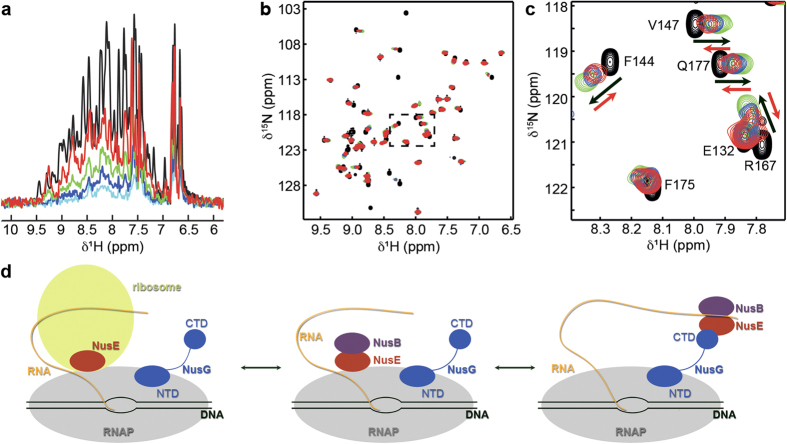
Competition of RNAP and NusG-CTD for NusE binding. (**a**) Displacement of RNAP from NusB:NusE^Δ^ by NusG-CTD. 1D [^1^H,^15^N]-HSQC spectra of free NusB:[^15^N]-NusE^Δ^, black, NusB:[^15^N]-NusE^Δ^ in the presence of RNAP in equimolar concentration, light blue, and NusB:[^15^N]-NusE^Δ^ in the presence of RNAP and NusG-CTD (molar ratio 1:1:1, dark blue; 1:1:3, green; 1:1:10, red). (**b**) Displacement of NusB:NusE^Δ^ from NusG-CTD by RNAP. 2D [^1^H,^15^N]-HSQC spectra of [^15^N]-NusG-CTD, black, [^15^N]-NusG-CTD in the presence of NusB:NusE^Δ^ in equimolar concentration, green, and [^15^N]-NusG-CTD in the presence of NusB:NusE^Δ^ and RNAP (molar ratio 1:1:1, blue; 1:1:3, red). (**c**) Detail of the rectangular region in (**b**). Black arrows indicate the chemical shift changes that occur upon addition of NusB:NusE^Δ^ to [^15^N]-NusG-CTD, red arrows show the changes upon subsequent addition of RNAP. (**d**) Schematic representation of the potential functions of a direct NusE:RNAP interaction. Color code as in Fig. 1.

Interestingly, NusE^Δ^’s binding surface for RNAP strongly overlaps with that for NusG-CTD so that binding of NusE^Δ^ to RNAP and NusG-CTD should be mutually exclusive (Fig. 3e)^14^. Thus we asked whether NusG-CTD and RNAP compete for binding to NusE. We performed a [^1^H,^15^N]-heteronuclear single quantum coherence (HSQC) displacement experiment in which the complex NusB:[^15^N]-NusE^Δ^:RNAP was titrated with NusG-CTD (Fig. 4a). In the one-dimensional (1D) [^1^H,^15^N]-HSQC spectra signals of [^15^N]-NusE^Δ^ strongly decreased upon NusB:[^15^N]-NusE^Δ^:RNAP complex formation as the increase of the molecular mass leads to significant line broadening. Titration with NusG-CTD reversed this effect, demonstrating the displacement of RNAP from NusB:[^15^N]-NusE^Δ^. The corresponding 2D [^1^H,^15^N]-HSQC spectra show that released NusB:[^15^N]-NusE^Δ^ binds to NusG-CTD (Supplementary Fig. 2). Thus, NusG-CTD can abstract NusE^Δ^ from RNAP. Next, we asked whether in reverse RNAP can displace NusG-CTD from the NusB:NusE^Δ^:NusG-CTD complex. We titrated NusB:NusE^Δ^:[^15^N]-NusG-CTD with RNAP and followed the titration by recording 2D [^1^H,^15^N]-HSQC spectra (Fig. 4b,c). Addition of NusB:NusE^Δ^ to [^15^N]-NusG-CTD led to changes in the chemical shifts of [^15^N]-NusG-CTD signals typical for NusB:NusE^Δ^:[^15^N]-NusG-CTD complex formation. Those changes were reversed by about 50% when RNAP was added in 3-fold molar excess, as expected on disruption of the NusB:NusE^Δ^:NusG-CTD complex by NusE:RNAP interaction. Thus, RNAP and NusG-CTD compete for NusE^Δ^ with similar low micromolar *K*_*D*_ values (NusB:NusE^Δ^:NusG-CTD: 50 μM)^14^.

These competition experiments support the notion of overlapping binding sites of NusE for NusG-CTD and RNAP, and they show that NusG-CTD can interact with NusE in the presence of RNAP. The complexes NusE:RNAP and NusE:NusG:RNAP *via* NusG are thus in a delicate equilibrium that can easily be influenced by other regulators such as transcription factors or certain RNA sequences. Overall, formation of the NusE:RNAP complex might play various roles during transcription (Fig. 4d). It might be involved either in transcription:translation coupling as the ribosome could directly contact RNAP *via* S10, e.g. when the RNA tether is relatively short, or in transcription antitermination where NusB:NusE is part of the antitermination complex^14^,^16^,^19^. The amount of free NusE that is not bound to the ribosome is estimated to be very low, but it is essential for transcription antitermination^36^. Thus tethering of NusE or the NusB:NusE complex to RNAP might be an early event in transcription antitermination to increase the local NusE concentration. NusE would remain bound to the TEC until transferred to NusG-CTD during assembly of the antitermination complex. As ribosomal operons comprise a very high density of transcribing RNAPs with high elongation rates^37^, tethering NusE directly to RNAP would ensure fast and efficient transcription antitermination in these operons.

### Solution structure of NusA-NTD from *E. coli*

The six domains comprising transcription factor NusA associates with RNAP *via* NusA-NTD, which is necessary and sufficient for the enhancement of pausing during transcription^27^. To determine the solution structure of NusA-NTD by NMR spectroscopy we initially tried a construct containing amino acids Met1-Ile137 carrying an N-terminal His_9_-tag, NusA(1–137). The high degree of heterogeneity in the peak intensities as well as the spectral overlap in the [^1^H,^15^N]-HSQC spectrum of the [^15^N]-labeled protein, however, prevented further analysis (Supplementary Fig. 3). A shorter construct, NusA-NTD^Δ^, consisting of amino acids Met1-Met125 and a cleavable C-terminal His_6_-tag, led to homogeneous signal intensities with non-overlapping signals in the [^1^H,^15^N]-HSQC spectra (Supplementary Fig. 3) and allowed nearly complete backbone and side chain resonance assignment. No resonances were found for residues Asp103, Arg104, Thr106, Thr107, and Gln108. These are located in a flexible loop so that severe line broadening may occur caused by either fast solvent exchange or conformational exchange on the intermediate chemical shift time scale. Structure determination was performed on the basis of 1565 distance and 193 dihedral restraints derived from multiple NMR experiments (Table 1).

**Figure 5:**
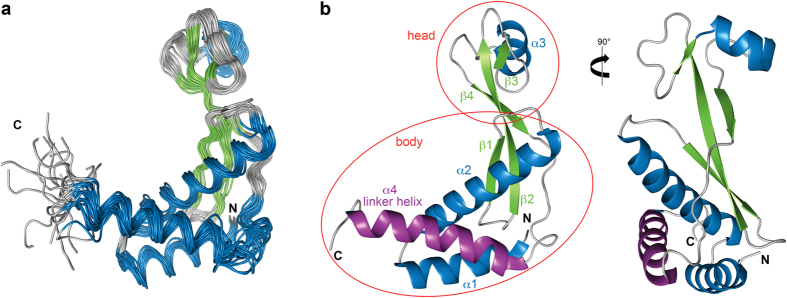
Solution structure of NusA-NTD^Δ^. (**a**) Structural ensemble of the 20 accepted lowest energy structures in ribbon representation colored according to secondary structure (α-helices, blue; β-strands, green; loops, grey). (**b**) Cartoon representation of the calculated structure with the lowest energy. Secondary structure elements are colored as in (**a**) and labeled. Helix α4 is highlighted in purple, the head and body parts are indicated.

NusA-NTD^Δ^ comprises four α-helices (α1: Asn2–Ala17, α2: Pro19–Glu40, α3: Leu77–Glu85, α4: Thr106–Ala124) and four β-strands (β1: Val45–Asp50, β2: Asp55–Val65, β3: Glu74–Thr76, β4: Gly90–Gln96) and its structure resembles that of NusA-NTDs from other bacteria^22^,^28^,^38^,^39^. It is L-shaped, with a globular head and a mainly α-helical body (Fig. 5a and b). In the latter α1, α2, α4, β1, and β2 surround an elongated hydrophobic core, and the long β2 strand protrudes into the globular head. The C-terminal helix α4 connects NusA-NTD and the NusA-SKK domain (linker helix). The globular head comprises α3, β3, β4, and the N-terminal part of β2. While the head is mainly acidic, the body exhibits large basic patches (Supplementary Fig. 4).

To date structures of NusA proteins from different bacteria are available, and although all NusA-NTDs are similar in their overall architecture, they differ in the position of the linker helix (Supplementary Fig. 5a–f). For NusA-NTD from *B. subtilis* (*Bs*NusA-NTD), NMR data suggest that this helix occurs in two alternative conformations in solution^28^. However, we have no indication for the presence of multiple conformations of helix α4 in NusA-NTD^Δ^. Moreover, unambiguous [^15^N]-nuclear Overhauser enhancement spectroscopy (NOESY) cross peaks between hydrophobic amino acids could be observed in NMR experiments, demonstrating a direct interaction between helix α4 and helices α1 and α2 in NusA-NTD^Δ^ (Supplementary Fig. 5g). As crystal structures of full length NusA from *Thermotoga maritima* (*Tm*NusA, protein data bank (PDB) IDs: 1HH2, 2L2F), *Mycobacterium tuberculosis* (*Mt*NusA, PDB ID: 1K0R) and *Planctomyces limnophilus* (*Pl*NusA, PDB ID: 4MTN) show that the NusA-SKK domain is connected to the linker helix by only a short loop, this helix might be responsible for the correct positioning of NusA-SKK for RNA binding.

Comparing NusA-NTD structures it is striking that *Mt*NusA-NTD and *Pl*NusA-NTD lack the globular head (Supplementary Fig. 5a–e), which is proposed to interact with the β′ subunit of RNAP^40^. This might indicate a different mode of action/binding of *Mt*NusA and *Pl*NusA compared to other NusAs.

### RNAP interface of NusA-NTD

**Figure 6:**
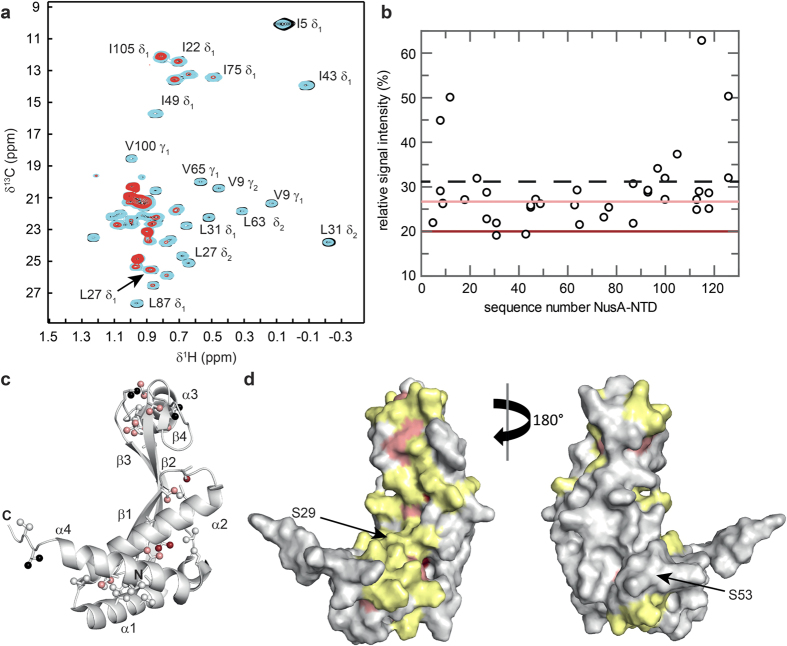
RNAP binding site of NusA-NTD^Δ^. (**a**) Titration of [I,L,V]-NusA-NTD^Δ^ with RNAP. Methyl-TROSY spectra of [I,L,V]-NusA-NTD^Δ^ in the absence, black, and in the presence of RNAP (1:1 molar ratio, cyan; 1:2 molar ratio, red), with assignment of representative signals. (**b**) Relative [I,L,V]-NusA-NTD^Δ^ signal intensity after addition of RNAP in equimolar concentration *vs*. amino acid sequence positions of NusA-NTD^Δ^. Dashed black line, average relative signal intensity; dark red and light red lines, thresholds for strongly affected (65% of the average relative intensity) and slightly affected (85% of the average relative intensity) residues, respectively. (**c**) Mapping of affected methyl groups onto the NusA-NTD^Δ^ structure. NusA-NTD^Δ^ (grey) in cartoon representation. Ile, Leu, and Val residues are in stick representation with the carbon atoms of their methyl groups as spheres. Strongly affected methyl groups, dark red; slightly affected methyl groups, light red; unaffected methyl groups, grey; unassigned methyl groups, black. (**d**) Mapping of affected residues onto the NusA-NTD^Δ^ structure (surface representation). For graphical illustration of the interaction site the complete amino acid was colored as affected in lieu of the methyl group. Colors are as in (**c**). Two amino acids on either side of an affected Ile/Leu/Val residue are highlighted in yellow unless they were unaffected Ile/Leu/Val residues. The positions of Ser29 and Ser53 are marked by black arrows.

NusA-NTD is supposed to bind to RNAP by interacting with the β flap tip helix of the β flap region, which forms the outer wall of the RNA exit channel. To date, available complex models are based on a low-resolution electron microscopy structure, cleavage experiments, targeted amino acid exchanges and NMR experiments using a short β flap construct^26^,^27^,^28^. Here we used complete RNAP to determine the RNAP binding site of NusA-NTD^Δ^ by applying the same approach as for NusG-NTD and NusE^Δ^. Methyl group labeled NusA-NTD^Δ^ ([I,L,V]-NusA-NTD^Δ^) was titrated with protonated RNAP leading to a non-uniform decrease of [I,L,V]-NusA-NTD^Δ^ methyl group signals (Fig. 6a). Again, the normalized signal intensity decrease in the 1:1 complex was analyzed to identify highly and slightly affected methyl groups (Fig. 6b). These are located mainly on the concave side of the body and in the acidic head (Fig. 6c). Inspection of the surface representation suggests that the β-sheet on the concave side of NusA-NTD^Δ^ is the center of the interaction surface, although it contains only a limited number of Ile, Leu, or Val residues resulting in a low structural resolution (Fig. 6d). Our binding site is in accordance with cleavage experiments using NusA variants NusA(S29C) and NusA(S53C), that indicated that S29 is located in the NusA:RNAP interface, while S53 is at the opposite side of NusA-NTD (Fig. 6d)^27^. Moreover, our results generally agree with mutational analyses showing that the concave side of the β-sheet is involved in NusA-NTD:β flap interaction^28^.

### Model of the NusA:RNAP complex

NusA has various effects on transcription elongation and termination with the NusA-NTD:RNAP interaction being probably one key step within the regulatory mechanism^27^. NusA-NTD contacts the RNA exit channel by binding to the β flap tip helix of the β flap region, but the resolution of the electron microscopy structure of a NusA-NTD:RNAP complex was too low to unambiguously determine the orientation of NusA-NTD bound to RNAP^26^. Cleavage and crosslinking experiments on the one hand and mutational analyses as well as NMR studies on *Bs*NusA-NTD and a short β flap construct on the other hand lead to two binding models^27^,^28^.

**Figure 7:**
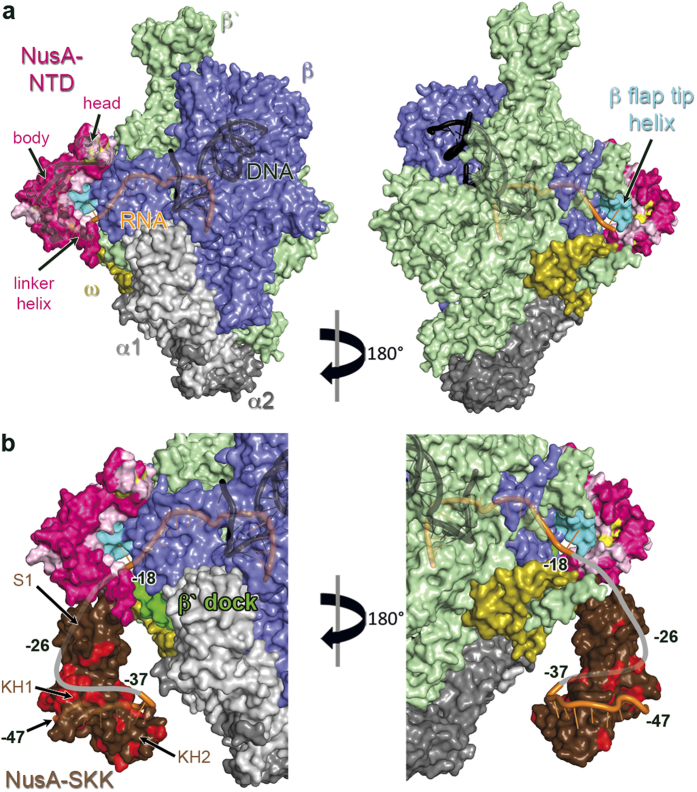
Model for the binding of NusA-NTD^Δ^ to elongating RNAP. (**a**) NusA-NTD^Δ^ (cartoon and surface representation, pink) is docked to elongating *Tt*RNAP (PDB ID: 2O5I, surface representation). Residues in NusA-NTD^Δ^ that are affected by RNAP binding are highlighted in yellow and two amino acids on either side of an affected Ile/Leu/Val residue are colored in light pink unless they were unaffected Ile/Leu/Val residues. α_1_, light grey; α_2_, dark grey; β, blue; β′, pale green; ω, olive; β flap tip helix, teal; RNA, orange; DNA, black. (**b**) Binding of exiting RNA by NusA. The orientation of NusA-NTD^Δ^ is the same as in (**a**), the position of *Tm*NusA-SKK was modeled by superposing *Tm*NusA-NTD (PDB ID: 1L2F) on NusA-NTD^Δ^. RNA was taken from the *Mt*NusA-SKK:RNA complex (PDB ID: 2ASB). Representation of NusA-NTD^Δ^, *Tt*RNAP and nucleic acids as in (**a**). The β′ dock domain is highlighted in green. *Tm*NusA-SKK (brown) is in surface representation with residues affected by RNA binding highlighted in red according to Schweimer *et al*.^4^. The grey line shows a possible path of exiting RNA, the estimated base numbers are indicated.

We used our NMR data to dock NusA-NTD^Δ^ to the β flap tip helix of elongating *Thermus thermophilus* RNAP (*Tt*RNAP, PDB ID: 2O5I) using HADDOCK^41^ (Fig. 7a). In the model most reliable according to HADDOCK, the body of NusA-NTD^Δ^ binds the β flap tip helix *via* its concave side, which is in accordance with other models^27^,^28^. The body is oriented towards the RNA exit channel so that the globular head interacts with the β′ subunit, the latter being in agreement with previous findings that the β′ subunit might also be involved in NusA-NTD binding^20^,^40^. This orientation allows a tight interaction with the *Tt*RNAP and is similar to the orientations suggested in earlier models^27^,^28^, although the absolute position of NusA-NTD^Δ^ strongly depends on the residues chosen as restraints and the position of the β flap tip helix.

Next, we integrated the NusA-SKK domain into the model (Fig. 7b). As the structure of *E. coli* NusA-SKK is not available and as the position of the linker helix is similar in *Pl*NusA and NusA-NTD^Δ^, we first used the crystal structure of *Pl*NusA as template. This, however, led to heavy steric clashes of the *Pl*NusA-SKK domain and *Tt*RNAP which could be prevented by rotating the *Pl*NusA-SKK domain away from the *Tt*RNAP, using the 3-4 residues following the linker helix as anchor. Alternatively, the linker helix itself might rotate slightly. Thus, we modeled the position of *Tm*NusA-SKK by superposing *Tm*NusA-NTD (PDB ID: 1L2F) on NusA-NTD^Δ^, and we added a short piece of RNA from the *Mt*NusA-SKK:RNA complex structure (PDB ID: 2ASB, Fig. 7b). Either way, the NusA-SKK domain can be positioned correctly for RNA binding. As NusA-NTD is necessary and sufficient for enhancing transcriptional pausing and recognizes duplex RNA^27^, exiting RNA might first contact a basic patch on the helical bundle of the NusA-NTD body (Supplementary Fig. 4), which is in direct vicinity of the RNAP exit channel. The RNA then wraps around the NusA-SKK domain, which, in turn, recognizes specific RNA signals (Fig. 7b)^4^,^42^,^43^. Crosslinking experiments showed that the RNA region −16 to −23 lies near the NusA-NTD in full-length NusA and that the −34 to −40 region of exiting RNA contacts the NusA-KH2 domain^27^, which is consistent with our model. Moreover, the NusA-S1 domain is placed in the vicinity of the β′ dock domain, being in accordance with a genetically shown NusA-S1:β′ dock interaction^44^ and cleavage experiments using Fe(III)-(*S*)-2-[4-(2-bromoacetamido)benzyl]ethylenediaminetetraacetic acid (FeBABE)^27^. The position of the C-terminus of NusA-SKK roughly orientates the two NusA-AR domains towards the α-subunits of RNAP and thus localizes NusA-AR2 close to the α-CTD, sterically simplifying a NusA-AR2:α-CTD interaction^4^.

Finally, it has been speculated that reorientation of helix α4 stabilizes RNA hairpins^28^. However, not only does NusA exhibit large conformational plasticity, but, in addition, the β flap tip helix is also a highly mobile element^28^. During the transcription cycle the flexibility of the β flap tip helix is important for the regulation of the size of the RNA exit channel, of which the β flap forms the outer wall. Thus, we suggest that the orientation of NusA-NTD bound to RNAP as well as the position of helix α4 may vary, depending on the position of the β flap tip helix. Moreover, this structural flexibility is complemented by the other NusA domains, which are all elastically connected.

### Outlook

In this conceptually simple single-experiment approach to identify the RNAP interaction surface of transcription factors with NMR spectroscopy (i) complete RNAP is used, (ii) probes in the transcription factor are directly monitored and, most importantly, (iii) none of the interaction partners needs to be modified. In the future, the method will be refined and used to study these interactions in more detail. Moreover, this approach is very general and can thus be transferred to other systems, with a small binding partner interacting with a supramolecular complex.

## Materials and Methods

### Cloning

The gene coding for *Ec*NusA-NTD(1–137) was cloned into pET19b *via Blp*I and *Bam*HI. The resulting *E. coli* expression vector pET19b_NusA-NTD_1-137 codes for a His_9_ tag fused to the N-terminus of NusA-NTD, cleavable by PreScission protease.

### Gene expression and protein purification

NusG-NTD was produced and purified as described^45^, as was NusA-NTD^Δ20^, the NusB:NusE^Δ^ complex^34^,^46^ and RNAP^20^.

Expression of *nusA-NTD(1–137)* was carried out in *E. coli* BL21 (λ DE3) (Novagen, Madison, WI, USA) harboring pET19b_NusA-NTD_1-137. Lysogeny broth (LB) medium supplemented with 100 μg/ml ampicillin was inoculated with a preculture to an optical density at 600 nm (*OD*_600_) of 0.2 and cells were grown at 37 °C until they reached an *OD*_600_ of 0.7. The temperature was lowered to 20 °C and 30 min later overexpression was induced with 2 mM IPTG. After overnight growth, cells were harvested by centrifugation (9,000 x *g*, 15 min, 4 °C) and dissolved in 20 mM tris(hydroxymethyl)aminomethane (Tris)/HCl (pH 7.9), 100 mM NaCl, 10% (v/v) glycerol, 5 mM β-mercaptoethanol, 10 mM imidazole (buffer A). Cell disruption was carried out with a microfluidizer (Microfluidics, Newton, MA, USA). Having centrifuged the lysate (12,000 × *g*, 30 min, 4 °C), the supernatant was applied to a Ni-NTA column (Qiagen, Hilden, Germany), and subsequently the column was washed with buffer A. A step gradient with increasing imidazole concentrations (10–500 mM in buffer A) was used for elution. Fractions containing His_9_-NusA-NTD(1–137) were combined and cleaved during overnight dialysis against 50 mM Tris/HCl (pH 8.0), 150 mM NaCl (molecular weight cut-off (MWCO) 3,500 Da) by PreScission protease (GE Healthcare, Munich, Germany). The protein solution was then dialyzed against 50 mM Tris (pH 7.4), 1 mM dithiothreitol (DTT, buffer B) and reapplied to the Ni-NTA column connected to a QXL FF column (GE Healthcare, Munich, Germany). After washing with buffer B, the Ni-NTA column was removed and the QXL FF column was eluted using a step gradient with increasing NaCl concentrations (0–1 M NaCl in buffer B). Fractions containing pure NusA-NTD(1-137) were dialyzed against the required buffer, concentrated by ultrafiltration (MWCO 3,000 Da) and stored at −80 °C after freezing with liquid nitrogen.

Proteins were uniformly labeled with ^15^N or ^15^N,^13^C by growing *E. coli* in M9 minimal medium^41^,^42^ with addition of (^15^NH_4_)_2_SO_4_ (Campro Scientific, Berlin, Germany) or (^15^NH_4_)_2_SO_4_ and ^13^C-D-glucose (Spectra Stable Isotopes, Columbia, MD, USA) as only nitrogen and carbon source. Expression and purification was the same as for proteins produced in LB medium. Methyl group labeling of Ile, Leu and Val residues with [^1^H,^13^C] in deuterated proteins was performed as described previously^20^.

### NMR spectroscopy

NMR spectroscopic experiments were conducted on Bruker *Avance* 600 MHz, 700 MHz and 800 MHz spectrometers, the latter two equipped with cryogenically cooled probes. For resonance assignment of NusA-NTD^Δ^, standard double and triple resonance through-bond experiments were recorded^47^,^48^. The protein was in 10 mM potassium phosphate buffer (pH 6.4) containing 50 mM NaCl at 298 K. NMR data were processed using in-house routines (Apodization, Fourier transformation, phase correction and baseline correction) and visualized with NMRView^49^. Distance restraints for structure calculation were derived from [^15^N]-edited and [^13^C]-edited NOESY spectra with mixing times of 100–120 ms. NOESY cross peaks were classified according to their relative intensities and converted to distance restraints with the following upper limits: 3.0 Å, strong; 4.0 Å, medium; 5.0 Å, weak; 6.0 Å, very weak. Experimental NOESY spectra were validated semi-quantitatively against back-calculated spectra to confirm the assignment and to avoid bias of upper distance restraints by spin-diffusion. Hydrogen bonds were included for backbone amide protons in regular secondary structure if the amide proton did not show a water exchange cross peak in the [^15^N]-edited NOESY spectrum. Backbone dihedral restraints were obtained from chemical shift data by using TALOS^50^. Existence of a hydrogen bond was assumed if the acceptor of a slowly exchanging amide proton, based on the absence of a water exchange peak in the [^15^N]-edited NOESY spectrum, could be identified unambiguously from the results of initial structure calculations. For each hydrogen bond the distance between the amide proton and the acceptor was restrained to less than 2.3 Å and the distance between the amide nitrogen and the acceptor to less than 3.1 Å.

The structure calculation was performed with the program XPLOR-NIH 2.1.2^51^ using a three-step simulated annealing protocol with floating assignment of prochiral groups including a conformational database potential^52^. For the final iteration 80 structures were calculated, the 20 structures of lowest energy were accepted and further analyzed with the programs XPLOR-NIH 2.1.2 and PROCHECK-NMR^53^.

TROSY spectra^29^ were recorded using [I,L,V]-labeled protein samples (20 μM) in 25 mM 4-(2-hydroxyethyl)-1-piperazineethanesulfonic acid (HEPES, pH 7.5), 50 mM NaCl, 5% (v/v) glycerol, 0.5 mM ethylenediaminetetraacetic acid (EDTA), 10 mM MgCl_2_, 10 μM ZnCl_2_, 1 mM DTT in 99.9% D_2_O at 298 K. Unlabeled, protonated RNAP in the same buffer was added in two steps (ratios 1:1, 1:2). Non-stereo-specific assignments of methyl groups of NusG-NTD and NusE^Δ^ were taken from previous studies^10^,^46^. Signal intensities were normalized by protein concentration and number of scans. As pulse lengths changed less than 1% upon RNAP addition, the influence of these changes on the intensity were neglected. For each titration step the ratio of remaining signal intensities and signal intensities in the spectrum of the free transcription factor were calculated, yielding relative signal intensities. Next, the mean value of all relative intensities in each titration step was determined and experiment-specific thresholds of the mean value were defined. Residues with relative signal intensities below these thresholds were classified as either strongly or slightly affected. Additionally, Leu and Val residues were considered as affected, when at least one of the two signals showed a significant intensity decrease. Only unambiguously assigned signals were used in the analysis.

Proteins for the displacement experiments of [^15^N]-NusE^Δ^:NusB from RNAP by NusG-CTD and of NusE^Δ^:NusB from [^15^N]-NusG-CTD by RNAP were in 25 mM HEPES, pH 7.5, 100 mM NaCl at 298 K. Separate samples for [^15^N]-NusE^Δ^:NusB (50 μM) and [^15^N]-NusE^Δ^:NusB:RNAP (25 μM each) were prepared. For the displacement experiments NusG-CTD was added (stock concentration: 1050 μM). Similarly, separate samples for [^15^N]-NusG-CTD (50 μM) and [^15^N]-NusG-CTD: NusE^Δ^:NusB (25 μM each) were prepared. For the displacement experiments RNAP was added from a 117 μM stock. The titrations were followed by recording 1D or 2D [^1^H,^15^N]-HSQC spectra after each titration step. 1D spectra were normalized by protein concentration and number of scans. As pulse lengths changed less than 1% upon RNAP addition, the influence of these changes on the intensity were neglected.

### Docking and Molecular Modeling

The NusG-NTD:RNAP complex was generated based on the crystal structure of Spt4/5 bound to the clamp domain from *P. furiosus* (PDB ID: 3QQC). *E. coli* NusG-NTD (PDB ID: 2K06, model 1) was superposed on Spt5 (chain D, root mean square deviation (r.m.s.d.) 1.2 Å). *Ec*RNAP (PDB ID: 4KMU) was positioned by superposing the β′ subunit (chain D) on the clamp domain (chain A, r.m.s.d. 2.4 Å).

Docking of NusA-NTD^Δ^ (model 1) to elongating *Tt*RNAP (PDB ID: 2O5I) was carried out using the HADDOCK webserver^41^. Residues in NusA-NTD^Δ^ that were experimentally determined to be affected by RNAP binding (Leu27, Leu31, Ile43, Val45) were defined as active residues. Solvent exposed residues in the β flap tip helix were chosen as active residues (chain C, residues Arg772, Leu773, Ser776, Ile777). Passive residues were automatically determined by HADDOCK. The coordinates of the β flap tip helix in the docked complex relative to the deposited coordinates of NusA-NTD^Δ^ are shown in Supplementary Table 1. After docking NusA-NTD^Δ^ to *Tt*RNAP, the position of the NusA-SKK domain was modeled with two alternative procedures. First, *Pl*NusA (PDB ID: 4MTN) was superposed on NusA-NTD^Δ^ (residues G3-D73 of *Pl*NusA; residues Met1-Thr101 of NusA-NTD^Δ^). To avoid clashes with *Tt*RNAP the *Pl*NusA-SKK was rotated manually around residues in the linker between *Pl*NusA-NTD and *Pl*NusA-SKK (residues Arg107-Gln109) using PyMOL^54^. In the second approach *Tm*NusA (PDB ID: 1L2F) was superposed on NusA-NTD^Δ^ using residues 1–101. Finally, the *Mt*NusA-SKK:RNA complex (PDB ID: 2ASB, residues Ser108-Gly333 of *Mt*NusA-SKK) was superposed on *Tm*NusA-SKK (residues Glu132-Leu344) to position the RNA. RNA base numbers were estimated.

### Programs

All structures were visualized with PyMOL^54^. The Adaptive Poisson-Boltzmann Solver (APBS)-Plugin and the PDB2PQR server were used for the determination of the charge surface potential^55^,^56^. Superpositions of different NusA-NTDs were done with LSQMAN^57^, omitting the linker helix (residues Met1-Thr101 of NusA-NTD^Δ^, residues Met1-Asn101 of *Tm*NusA (PDB ID: 1L2F, 1HH2), residues Met1-Asp101 of *Bs*NusA (PDB ID: 2MT4), residues Met1-Phe79 of *Mt*NusA (PDB ID: 2K0R), residues Gly3-Asp73 of *Pl*NusA (PDB ID: 4MTN)). All other superpositions were carried out by PyMOL^54^.

## Additional Information

**Accession codes:** Chemical shifts of NusA-NTD^Δ^ have been deposited in the Biological Magnetic Resonance Bank Databank, accession number 16868. The atomic coordinates of the NusA-NTD^Δ^ structure have been deposited in the Protein Data Bank, accession number 2KWP. 

**How to cite this article**: Drögemüller, J. *et al*. Determination of RNA polymerase binding surfaces of transcription factors by NMR spectroscopy. *Sci. Rep*. **5**, 16428; doi: 10.1038/srep16428 (2015).

## Supplementary Material

Supplementary Information

## Figures and Tables

**Figure 1:**
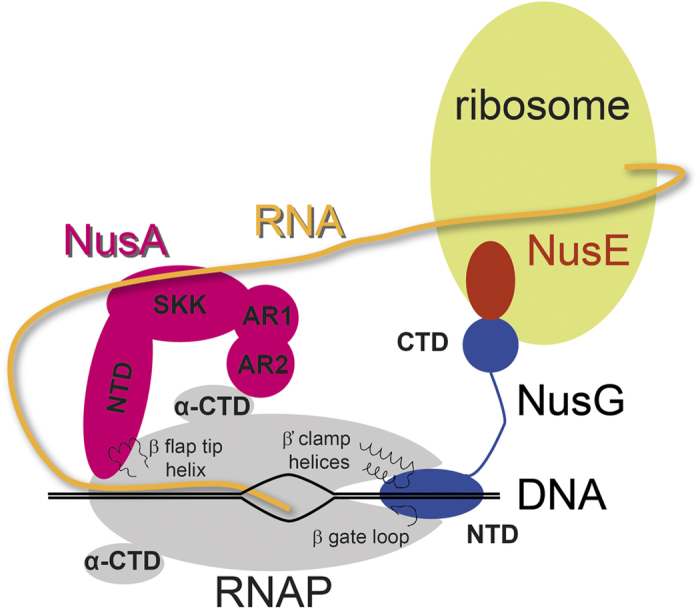
Schematic representation of transcription:translation coupling. NusA, pink, NusE, red; NusG, blue; RNAP, grey; ribosome, light green; DNA, black; RNA, yellow. In RNAP selected structural elements involved in Nus factor binding are indicated.

**Table 1:** Experimental constraints for structure calculation of NusA-NTD^Δ^.

Distance restraints	total	1507
	intraresidual	329
	sequential	386
	medium range	321
	long range	471
Hydrogen bond restraints		58
Dihedral restraints		193
Restraint violations	rms distance violation (Å)	0.006 (±0.0011)
	max. distance violation (Å)	0.11
	rms dihedral violation (°)	0.05 (±0.02)
	max. dihedral violation (°)	0.8
	rmsd bond length (Å)	0.00070 (±0.00009)
	rmsd bond angle (°)	0.13 (±0.012)
Atomic coordinate precision	backbone atoms (Å)	0.80^a^
	all heavy atoms (Å)	1.13^a^
Ramachandran plot statistics^b^	most favored regions (%)	90.5
	additional allowed regions (%)	8.8
	generously allowed regions (%)	0.2
	disallowed regions (%)	0.5

^a^residues Met1-Arg123.

^b^determined by PROCHECK-NMR.
