# Supplementary material for: Determination of RNA polymerase binding surfaces of transcription factors by NMR spectroscopy
Source: Sci Rep. 2015 Nov 12;5:16428. doi: 10.1038/srep16428 (PMC4642336; doi:10.1038/srep16428)
Supplement: Supplementary Information [file srep16428-s1.pdf]

Supplementary Information

Determination of RNA polymerase binding surfaces of transcription factors by NMR spectroscopy

Johanna Drögemüller[1,§], Martin Strauß[1,§], Kristian Schweimer[1], Marcel Jurk[2], Paul Rösch[1],

Stefan H Knauer[1,*]

[1] Lehrstuhl Biopolymere und Forschungszentrum für Bio-Makromoleküle, Universität Bayreuth, Universitätsstraße 30, 95447 Bayreuth, Germany

[2] present address: Max Planck Institute for Molecular Genetics, Ihnestr. 63-73, 14195 Berlin, Germany

[§] These authors contributed equally to this work

* corresponding author

Contents

Supplementary Table 1 2

Supplementary Figure 1 4

Supplementary Figure 2 5

Supplementary Figure 3 6

Supplementary Figure 4 7

Supplementary Figure 5 8

Supplementary References 10

Supplementary Table 1: Coordinates of the β flap tip helix in the modeled NusA-NTD$^{\Delta}$:*Tt*RNAP complex. The table is an extract of the PDB file of elongating *Tt*RNAP (residues 767-781 of the β subunit; PDB ID: 2O5I) docked to NusA-NTD$^{\Delta}$ as described in the Material and Methods section, giving the position of *Tt*RNAP relative to the deposited coordinates of NusA-NTD$^{\Delta}$ (PDB ID: 2KWP).

```
ATOM  10777  N   PRO C 767       3.453  -1.849 -42.571  1.00 90.52           N
ATOM  10778  CA  PRO C 767       2.398  -2.073 -41.584  1.00 91.06           C
ATOM  10779  C   PRO C 767       2.633  -1.165 -40.362  1.00 92.21           C
ATOM  10780  O   PRO C 767       3.412  -0.213 -40.472  1.00 92.68           O
ATOM  10781  CB  PRO C 767       2.524  -3.559 -41.274  1.00 90.38           C
ATOM  10782  CG  PRO C 767       3.028  -4.129 -42.533  1.00 90.03           C
ATOM  10783  CD  PRO C 767       4.098  -3.134 -42.897  1.00 90.44           C
ATOM  10784  N   THR C 768       2.013  -1.418 -39.212  1.00 92.37           N
ATOM  10785  CA  THR C 768       2.261  -0.517 -38.082  1.00 93.42           C
ATOM  10786  C   THR C 768       2.739  -1.281 -36.849  1.00 94.34           C
ATOM  10787  O   THR C 768       2.104  -2.254 -36.442  1.00 94.97           O
ATOM  10788  CB  THR C 768       0.994   0.286 -37.704  1.00 92.74           C
ATOM  10789  OG1 THR C 768       0.329   0.726 -38.894  1.00 92.51           O
ATOM  10790  CG2 THR C 768       1.369   1.533 -36.866  1.00 91.14           C
ATOM  10791  N   PRO C 769       3.867  -0.844 -36.239  1.00 95.40           N
ATOM  10792  CA  PRO C 769       4.475  -1.458 -35.040  1.00 95.44           C
ATOM  10793  C   PRO C 769       3.577  -1.368 -33.769  1.00 95.51           C
ATOM  10794  O   PRO C 769       3.583  -2.294 -32.943  1.00 95.59           O
ATOM  10795  CB  PRO C 769       5.803  -0.706 -34.892  1.00 96.45           C
ATOM  10796  CG  PRO C 769       6.147  -0.342 -36.332  1.00 95.78           C
ATOM  10797  CD  PRO C 769       4.806   0.125 -36.849  1.00 95.78           C
ATOM  10798  N   GLU C 770       2.827  -0.273 -33.609  1.00 93.98           N
ATOM  10799  CA  GLU C 770       1.962  -0.077 -32.434  1.00 93.34           C
ATOM  10800  C   GLU C 770       0.563  -0.681 -32.666  1.00 93.16           C
ATOM  10801  O   GLU C 770      -0.118  -1.081 -31.718  1.00 92.30           O
ATOM  10802  CB  GLU C 770       1.849   1.427 -32.127  1.00 92.37           C
ATOM  10803  CG  GLU C 770       3.152   2.249 -32.385  1.00 90.19           C
ATOM  10804  CD  GLU C 770       3.957   2.594 -31.128  1.00 88.39           C
ATOM  10805  OE1 GLU C 770       4.437   1.679 -30.437  1.00 87.21           O
ATOM  10806  OE2 GLU C 770       4.113   3.798 -30.832  1.00 87.50           O
ATOM  10807  N   GLU C 771       0.159  -0.730 -33.938  1.00 93.66           N
ATOM  10808  CA  GLU C 771      -1.116  -1.314 -34.376  1.00 95.22           C
ATOM  10809  C   GLU C 771      -0.991  -2.838 -34.494  1.00 96.29           C
ATOM  10810  O   GLU C 771      -1.993  -3.557 -34.560  1.00 96.81           O
ATOM  10811  CB  GLU C 771      -1.531  -0.719 -35.743  1.00 95.59           C
ATOM  10812  CG  GLU C 771      -2.798  -1.302 -36.423  1.00 95.80           C
ATOM  10813  CD  GLU C 771      -2.986  -0.867 -37.893  1.00 96.05           C
ATOM  10814  OE1 GLU C 771      -4.129  -0.944 -38.403  1.00 95.71           O
ATOM  10815  OE2 GLU C 771      -1.999  -0.457 -38.544  1.00 95.66           O
ATOM  10816  N   ARG C 772       0.255  -3.318 -34.526  1.00 95.94           N
ATOM  10817  CA  ARG C 772       0.562  -4.755 -34.598  1.00 96.21           C
ATOM  10818  C   ARG C 772       0.310  -5.431 -33.247  1.00 97.11           C
ATOM  10819  O   ARG C 772      -0.155  -6.577 -33.193  1.00 97.59           O
ATOM  10820  CB  ARG C 772       2.031  -4.975 -34.992  1.00 94.95           C
ATOM  10821  CG  ARG C 772       2.497  -6.441 -35.071  1.00 92.48           C
ATOM  10822  CD  ARG C 772       1.926  -7.149 -36.290  1.00 91.56           C
ATOM  10823  NE  ARG C 772       1.943  -6.309 -37.490  1.00 90.45           N
ATOM  10824  CZ  ARG C 772       1.929  -6.773 -38.739  1.00 90.61           C
ATOM  10825  NH1 ARG C 772       1.906  -8.079 -38.978  1.00 91.27           N
ATOM  10826  NH2 ARG C 772       1.936  -5.935 -39.759  1.00 89.96           N
ATOM  10827  N   LEU C 773       0.629  -4.710 -32.164  1.00 97.02           N
ATOM  10828  CA  LEU C 773       0.423  -5.199 -30.800  1.00 97.16           C
ATOM  10829  C   LEU C 773      -1.069  -5.199 -30.435  1.00 96.83           C
ATOM  10830  O   LEU C 773      -1.557  -6.174 -29.874  1.00 97.78           O
ATOM  10831  CB  LEU C 773       1.212  -4.334 -29.791  1.00 96.99           C
ATOM  10832  CG  LEU C 773       2.759  -4.388 -29.822  1.00 96.94           C
```

ATOM	10833	CD1	LEU	C	773	3.329	-3.401	-28.800	1.00	96.92	C
ATOM	10834	CD2	LEU	C	773	3.246	-5.800	-29.502	1.00	97.02	C
ATOM	10835	N	LEU	C	774	-1.789	-4.120	-30.755	1.00	95.96	N
ATOM	10836	CA	LEU	C	774	-3.229	-4.033	-30.473	1.00	95.46	C
ATOM	10837	C	LEU	C	774	-3.998	-5.145	-31.174	1.00	95.47	C
ATOM	10838	O	LEU	C	774	-4.830	-5.817	-30.562	1.00	96.22	O
ATOM	10839	CB	LEU	C	774	-3.806	-2.692	-30.942	1.00	93.90	C
ATOM	10840	CG	LEU	C	774	-5.346	-2.636	-30.934	1.00	92.29	C
ATOM	10841	CD1	LEU	C	774	-5.830	-2.770	-29.504	1.00	91.81	C
ATOM	10842	CD2	LEU	C	774	-5.833	-1.345	-31.545	1.00	91.22	C
ATOM	10843	N	ARG	C	775	-3.735	-5.325	-32.462	1.00	95.29	N
ATOM	10844	CA	ARG	C	775	-4.402	-6.381	-33.208	1.00	94.68	C
ATOM	10845	C	ARG	C	775	-4.209	-7.740	-32.520	1.00	94.67	C
ATOM	10846	O	ARG	C	775	-5.184	-8.404	-32.214	1.00	94.44	O
ATOM	10847	CB	ARG	C	775	-3.860	-6.445	-34.654	1.00	94.56	C
ATOM	10848	CG	ARG	C	775	-4.360	-5.341	-35.613	1.00	92.12	C
ATOM	10849	CD	ARG	C	775	-5.880	-5.356	-35.749	1.00	90.64	C
ATOM	10850	NE	ARG	C	775	-6.342	-4.568	-36.888	1.00	89.53	N
ATOM	10851	CZ	ARG	C	775	-7.616	-4.420	-37.234	1.00	88.70	C
ATOM	10852	NH1	ARG	C	775	-8.567	-5.009	-36.526	1.00	88.08	N
ATOM	10853	NH2	ARG	C	775	-7.937	-3.684	-38.292	1.00	88.26	N
ATOM	10854	N	SER	C	776	-2.950	-8.120	-32.264	1.00	94.65	N
ATOM	10855	CA	SER	C	776	-2.568	-9.411	-31.644	1.00	94.82	C
ATOM	10856	C	SER	C	776	-3.212	-9.627	-30.236	1.00	94.23	C
ATOM	10857	O	SER	C	776	-3.590	-10.761	-29.910	1.00	94.33	O
ATOM	10858	CB	SER	C	776	-1.021	-9.512	-31.521	1.00	94.77	C
ATOM	10859	OG	SER	C	776	-0.360	-9.645	-32.780	1.00	94.97	O
ATOM	10860	N	ILE	C	777	-3.334	-8.580	-29.415	1.00	93.70	N
ATOM	10861	CA	ILE	C	777	-3.901	-8.698	-28.057	1.00	92.75	C
ATOM	10862	C	ILE	C	777	-5.350	-9.252	-28.072	1.00	93.16	C
ATOM	10863	O	ILE	C	777	-5.631	-10.255	-27.404	1.00	93.55	O
ATOM	10864	CB	ILE	C	777	-3.896	-7.308	-27.305	1.00	92.10	C
ATOM	10865	CG1	ILE	C	777	-2.455	-6.821	-27.095	1.00	91.21	C
ATOM	10866	CG2	ILE	C	777	-4.534	-7.449	-25.920	1.00	91.63	C
ATOM	10867	CD1	ILE	C	777	-2.357	-5.456	-26.433	1.00	90.12	C
ATOM	10868	N	PHE	C	778	-6.256	-8.615	-28.820	1.00	93.48	N
ATOM	10869	CA	PHE	C	778	-7.673	-9.029	-28.887	1.00	93.33	C
ATOM	10870	C	PHE	C	778	-7.928	-10.029	-30.029	1.00	94.16	C
ATOM	10871	O	PHE	C	778	-8.818	-10.872	-29.934	1.00	93.84	O
ATOM	10872	CB	PHE	C	778	-8.583	-7.804	-29.105	1.00	92.40	C
ATOM	10873	CG	PHE	C	778	-8.687	-6.861	-27.918	1.00	91.76	C
ATOM	10874	CD1	PHE	C	778	-7.540	-6.454	-27.198	1.00	91.29	C
ATOM	10875	CD2	PHE	C	778	-9.943	-6.350	-27.533	1.00	91.20	C
ATOM	10876	CE1	PHE	C	778	-7.638	-5.546	-26.104	1.00	90.92	C
ATOM	10877	CE2	PHE	C	778	-10.067	-5.440	-26.441	1.00	91.24	C
ATOM	10878	CZ	PHE	C	778	-8.906	-5.037	-25.723	1.00	90.85	C
ATOM	10879	N	GLY	C	779	-7.153	-9.925	-31.108	1.00	95.41	N
ATOM	10880	CA	GLY	C	779	-7.313	-10.826	-32.242	1.00	97.01	C
ATOM	10881	C	GLY	C	779	-6.406	-10.473	-33.421	1.00	97.94	C
ATOM	10882	O	GLY	C	779	-6.809	-9.697	-34.309	1.00	98.21	O
ATOM	10883	N	GLU	C	780	-5.190	-11.038	-33.414	1.00	98.62	N
ATOM	10884	CA	GLU	C	780	-4.154	-10.817	-34.448	1.00	98.20	C
ATOM	10885	C	GLU	C	780	-4.735	-10.916	-35.859	1.00	97.77	C
ATOM	10886	O	GLU	C	780	-4.483	-10.050	-36.707	1.00	97.44	O
ATOM	10887	CB	GLU	C	780	-3.002	-11.842	-34.299	1.00	98.74	C
ATOM	10888	CG	GLU	C	780	-3.479	-13.315	-34.185	1.00	98.09	C
ATOM	10889	CD	GLU	C	780	-2.821	-14.257	-35.186	1.00	97.77	C
ATOM	10890	OE1	GLU	C	780	-3.289	-15.415	-35.292	1.00	97.28	O
ATOM	10891	OE2	GLU	C	780	-1.848	-13.846	-35.859	1.00	97.54	O
ATOM	10892	N	LYS	C	781	-5.507	-11.978	-36.099	1.00	97.33	N
ATOM	10893	CA	LYS	C	781	-6.147	-12.202	-37.392	1.00	96.31	C
ATOM	10894	C	LYS	C	781	-7.292	-11.208	-37.620	1.00	95.87	C
ATOM	10895	O	LYS	C	781	-8.485	-11.533	-37.511	1.00	96.54	O
ATOM	10896	CB	LYS	C	781	-6.675	-13.642	-37.491	1.00	95.28	C
ATOM	10897	CG	LYS	C	781	-7.331	-14.173	-36.226	1.00	93.86	C
ATOM	10898	CD	LYS	C	781	-8.176	-15.409	-36.492	1.00	93.01	C
ATOM	10899	CE	LYS	C	781	-7.389	-16.500	-37.197	1.00	92.99	C
ATOM	10900	NZ	LYS	C	781	-6.056	-16.712	-36.568	1.00	93.33	N

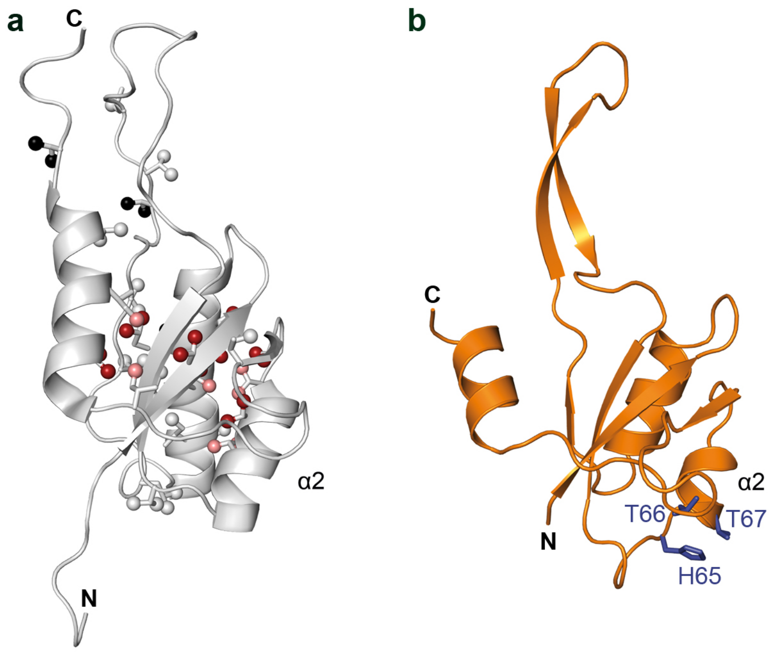

Supplementary Figure 1: Structures of NusG-NTD and RfaH-NTD. (**a**) RNAP binding site of NusG-NTD. Structure of NusG-NTD (PDB ID: 2K06) in cartoon representation, grey. Ile, Leu, and Val residues are shown as sticks with the carbon atoms of their methyl groups represented as spheres. Strongly affected methyl groups, dark red; slightly affected methyl groups, light red; unaffected methyl groups, grey; unassigned methyl groups, black. (**b**) βGL binding motif of RfaH-NTD. Structure of RfaH-NTD (PDB ID: 2OUG) in cartoon representation, orange. Residues involved in βGL binding are shown as blue sticks and labeled.

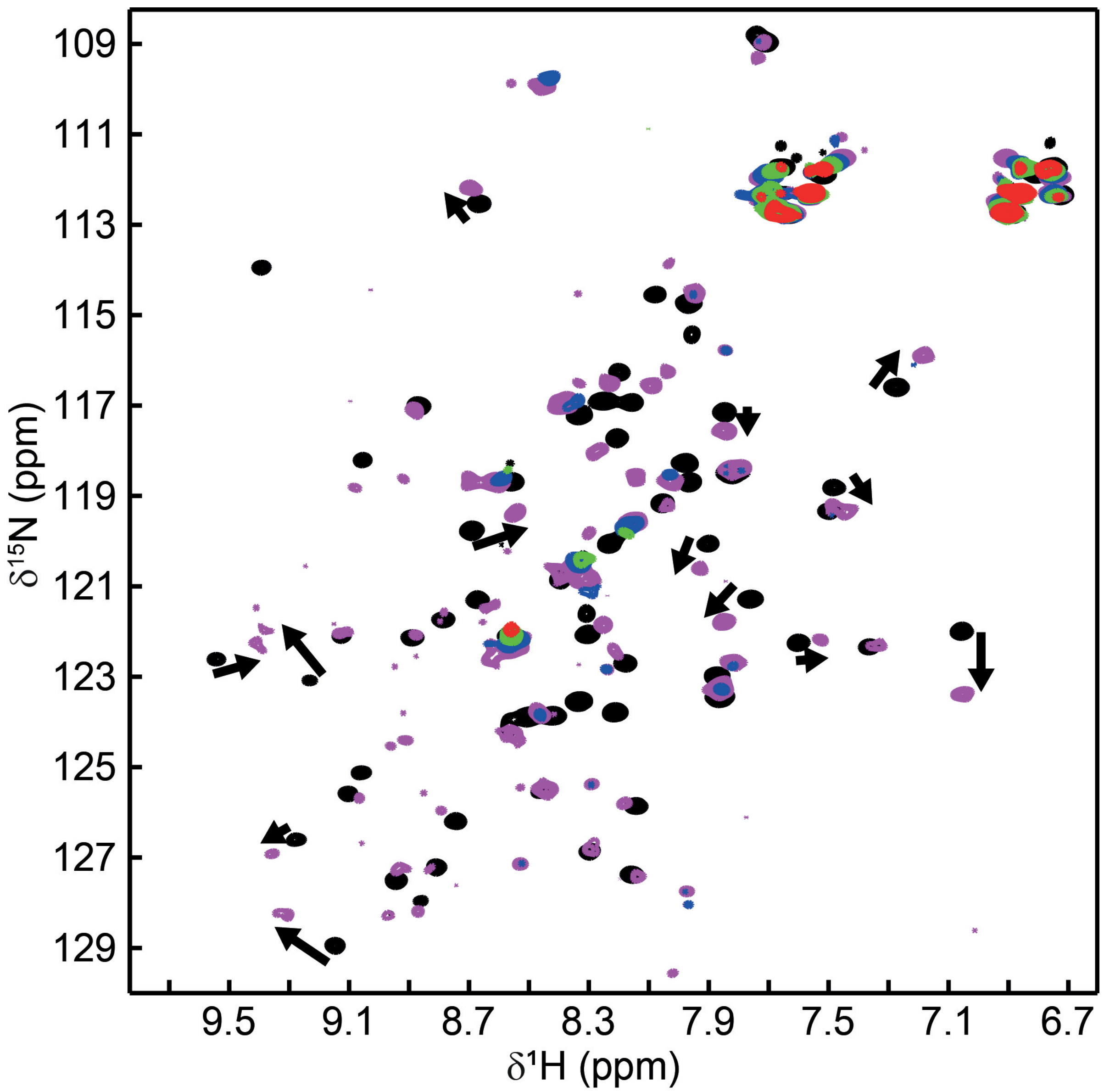

Supplementary Figure 2: Displacement of RNAP from NusE$^{\Delta}$ by NusG-CTD. 2D [^{1}H,^{15}N]-HSQC spectra of free NusB:[^{15}N]-NusE$^{\Delta}$, black, NusB:[^{15}N]-NusE$^{\Delta}$ in the presence of RNAP in equimolar concentration, red, and NusB:[^{15}N]-NusE$^{\Delta}$ in the presence of RNAP and NusG-CTD (molar ratio 1:1:1, green; 1:1:3, blue; 1:1:10, purple). Black arrows indicate the chemical shift changes that occur upon complex formation of NusG-CTD and NusB:[^{15}N]-NusE$^{\Delta1}$.

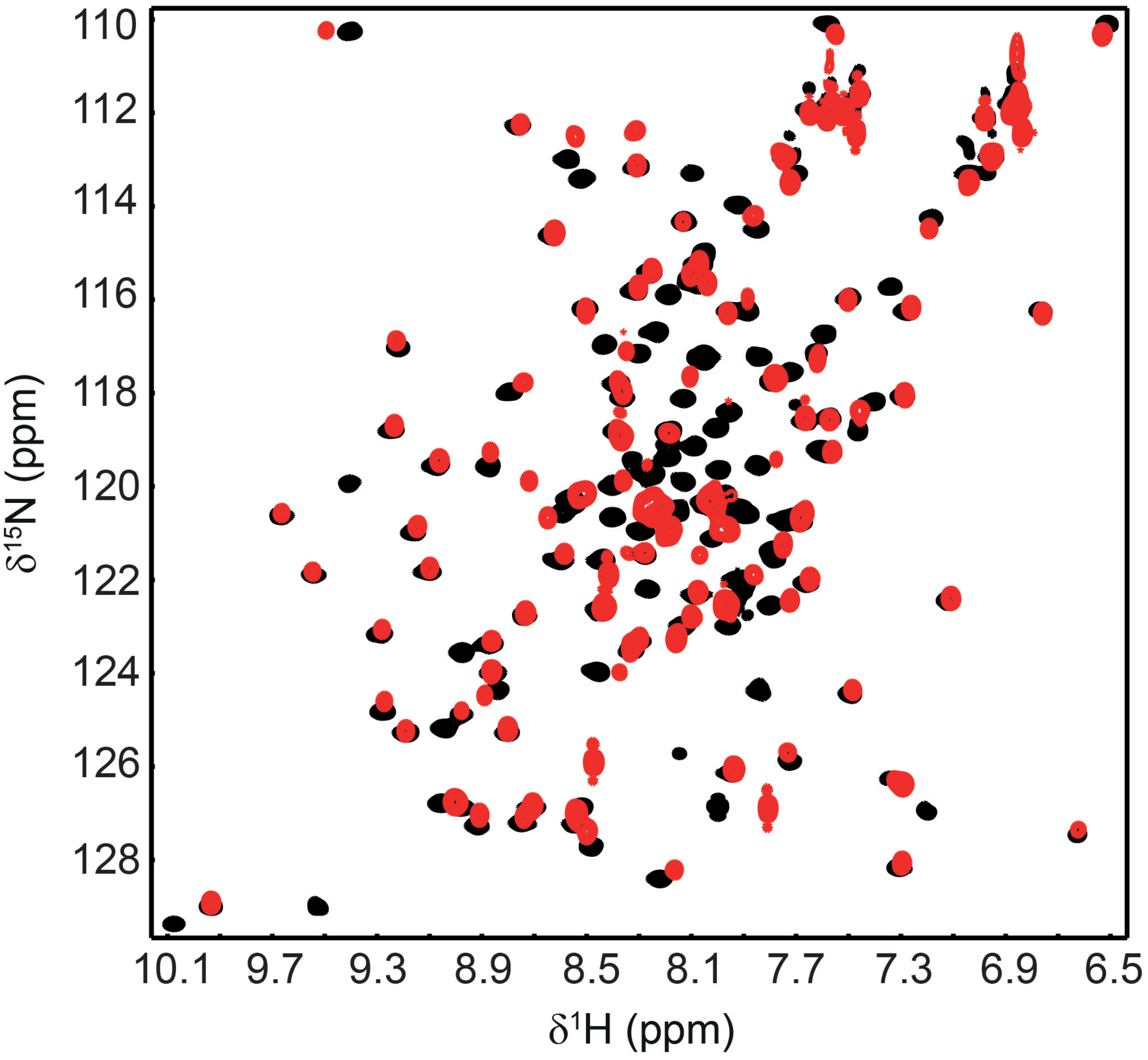

Supplementary Figure 3: Superposition of the [^{1}H,^{15}N]-HSQC spectra of [^{15}N]-NusA-NTD(1-137), red, and [^{15}N]-NusA-NTD$^{\Delta}$, black. The protein concentration was 400 µM in each sample.

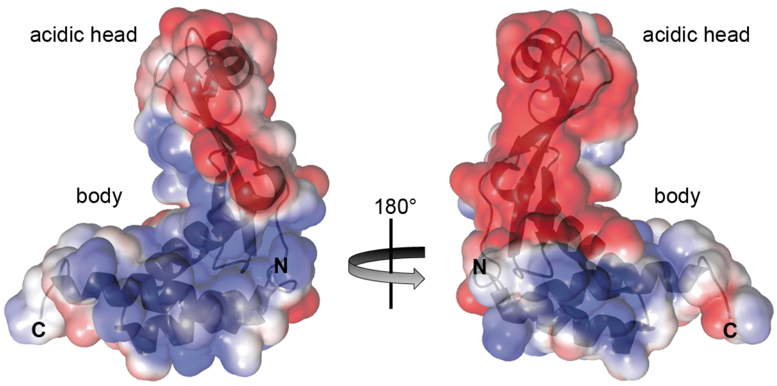

Supplementary Figure 4: Electrostatic potential molecular surface of NusA-NTD$^{\Delta}$. NusA-NTD$^{\Delta}$ in cartoon and surface representation. The electrostatic surface potential is colored from -2 kT/e, red, to +2 kT/e, blue.

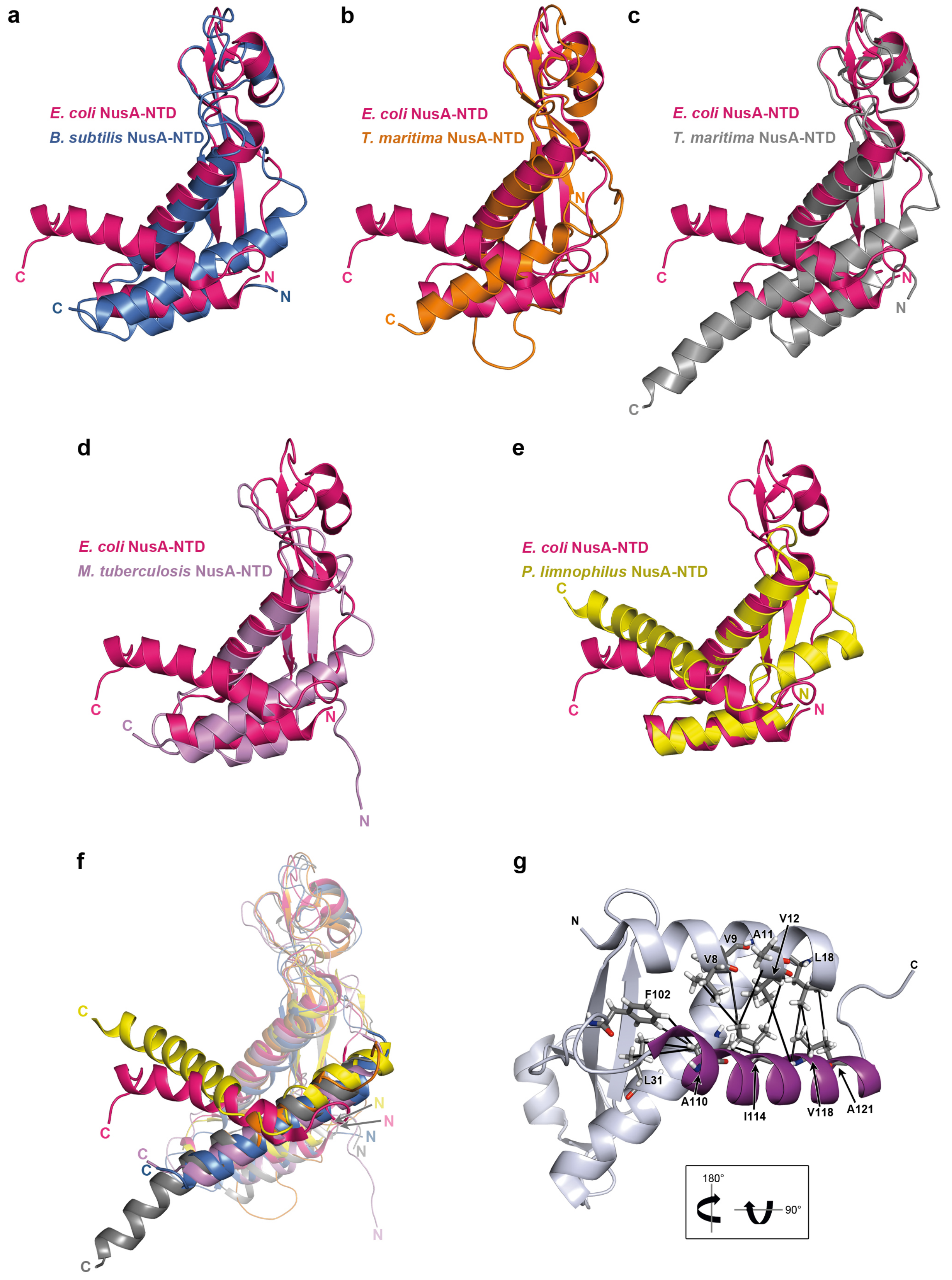

Supplementary Figure 5: Comparison of NusA-NTD structures. (**a-e**) Superposition of NusA-NTD$^{\Delta}$ (pink) with (**a**) *Bs*NusA-NTD (blue, PDB ID: 2MT4, root mean square deviation

(r.m.s.d.) 1.8 Å), (**b**) *Tm*NusA-NTD (orange, PDB ID: 1HH2, r.m.s.d. 1.9 Å) (**c**) *Tm*NusA-NTD (grey, PDB ID: 1L2F, r.m.s.d. 1.7 Å), (**d**) *Mt*NusA-NTD (violet, PDB ID: 1K0R, r.m.s.d. 1.8 Å), and (**e**) *Pl*NusA-NTD (yellow, PDB ID: 4MTN, r.m.s.d. 1.4 Å). The linker helix was not used for the superpositions. **(f)** Superposition of NusA-NTD structures shown in (**a-e**). The linker helix is shown in bright colors. **(g)** NOE network fixing the position of the linker helix in NusA-NTD$^{\Delta}$ (cartoon representation, grey; the linker helix is highlighted in purple). The inset indicates how the molecule is rotated in respect to (**a**). Residues participating in the NOE network are labeled and shown as sticks (carbon atoms, dark grey; nitrogen atoms, blue; oxygen atoms, red; hydrogen atoms, white). Unambiguously identified NOEs are shown as black lines. For clarity only one NOE is displayed per methyl group (using the corresponding methyl carbon atom as center).

Supplementary References

1. Burmann, B. M. *et al*. A NusE:NusG Complex Links Transcription and Translation. *Science* **328**, 501-504 (2010).
